# Advancing Vaccine Strategies against *Candida* Infections: Exploring New Frontiers

**DOI:** 10.3390/vaccines11111658

**Published:** 2023-10-29

**Authors:** Gurpreet Kaur, Sonam Chawla, Piyush Kumar, Ritu Singh

**Affiliations:** 1 Department of Biotechnology, Chandigarh College of Technology (CCT), Chandigarh Group of Colleges (CGC), Landran, Mohali 140307, India; 2 Department of Biotechnology, Jaypee Institute of Information Technology, Sector 62, Noida 201309, India; contact.schawla@gmail.com (S.C.);

**Keywords:** fungal immunity, *Candida*, candidiasis prevention, therapeutic innovations, antifungal vaccines, opportunistic infections

## Abstract

*Candida albicans*, along with several non-albicans *Candida* species, comprise a prominent fungal pathogen in humans, leading to candidiasis in various organs. The global impact of candidiasis in terms of disease burden, suffering, and fatalities is alarmingly high, making it a pressing global healthcare concern. Current treatment options rely on antifungal drugs such as azoles, polyenes, and echinocandins but are delimited due to the emergence of drug-resistant strains and associated adverse effects. The current review highlights the striking absence of a licensed antifungal vaccine for human use and the urgent need to shift our focus toward developing an anti-Candida vaccine. A number of factors affect the development of vaccines against fungal infections, including the host, intraspecies and interspecies antigenic variations, and hence, a lack of commercial interest. In addition, individuals with a high risk of fungal infection tend to be immunocompromised, so they are less likely to respond to inactivated or subunit whole organisms. Therefore, it is pertinent to discover newer and novel alternative strategies to develop safe and effective vaccines against fungal infections. This review article provides an overview of current vaccination strategies (live attenuated, whole-cell killed, subunit, conjugate, and oral vaccine), including their preclinical and clinical data on efficacy and safety. We also discuss the mechanisms of immune protection against candidiasis, including the role of innate and adaptive immunity and potential biomarkers of protection. Challenges, solutions, and future directions in vaccine development, namely, exploring novel adjuvants, harnessing the trained immunity, and utilizing immunoinformatics approaches for vaccine design and development, are also discussed. This review concludes with a summary of key findings, their implications for clinical practice and public health, and a call to action for continued investment in candidiasis vaccine research.

## 1. Introduction

### 1.1. Brief Overview of Candidiasis and Its Global Burden

Throughout the annals of medical history, the *Candida* species has undergone a metamorphic evolution, transcending from inconspicuous pathogens deemed as mere nuisances to potent and pervasive adversaries responsible for severe human ailments.

**Figure 1:**
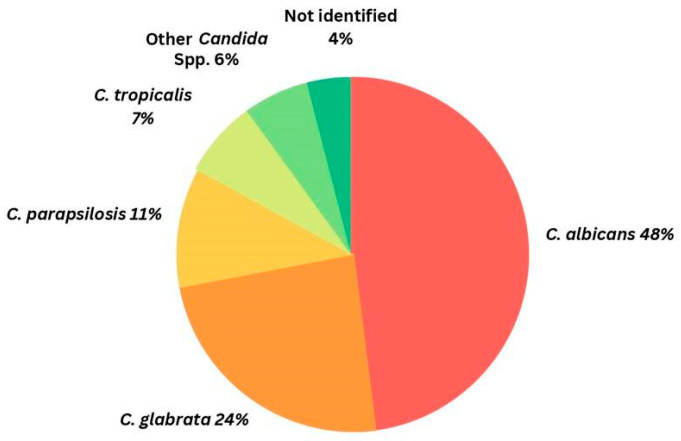
Species distribution for invasive candidiasis per 100,000 cases from 2009–2017.

Candidiasis, a well-recognized mycosis, encompasses a spectrum of cutaneous, mucosal, and organ infections that emerge at different life stages and are often associated with identifiable predisposing factors [1]. Central to this condition is the colonization of *Candida* spp., indispensable commensal yeasts found on human skin, in the gut, and in the genitourinary tract in up to 60% of healthy individuals, as well as at sites other than the ones enlisted [1]. This infectious colonization poses a grave health risk to immunocompromised individuals. *Candida albicans* stands out as the primary pathogen responsible for candidiasis; however, other species collectively named as non-albicans species—*C. glabrata*, *C. parapsilosis*, *C. tropicalis*, and *C. krusei*—are also now gaining medical attention (Figure 1) [2,3]. Taxonomic revisions have led to the renaming of several pathogenic *Candida* species, but the term candidiasis still encompasses infections caused by these agents [4]. Morphologically, *Candida* presents as small, oval, thin-walled yeast-like fungi, propagating through budding or fission [5]. An essential feature of these fungi is their non-photosynthetic, eukaryotic nature, possessing an external cell wall adjacent to the plasma membrane, and abundant sterols, notably ergosterol, in the plasma membrane [6].

While superficial infections primarily acquired within the community, result in significant morbidity, deep-seated and systemic *Candida* infections more commonly originate in healthcare settings. Notably, *C. albicans*, *C. glabrata*, *C. parapsilosis*, *C. tropicalis*, and *C. krusei* account for more than 90% of invasive infections [5].

Evidently, there is an escalating global burden of candidiasis which presents a dire health crisis, with the number of cases on the rise due to the growing population of immunocompromised individuals [7,8]. Invasive candidiasis, including both bloodstream and deep tissue-seated candidiasis, is the prevailing fungal ailment encountered within hospitals in high-income nations, with a global prevalence spanning from approximately 250,000 to 700,000 individuals annually. Its incidence rate falls within the range of 2 to 14 cases per 100,000 persons, while mortality rates vary from 40% to 55% [9]. It is important to note that global annual incidence rates cannot be reliably determined due to the limited availability of comprehensive studies. This increasing burden is matched by none other than tuberculosis; however, the domain of anti-*Candida* interventions is still in its infancy [8,10]. The United States incurs medical expenses exceeding USD 7.2 billion annually due to fungal diseases [7]. Moreover, the emergence of *Candida auris*, a new and highly virulent *Candida* species, has added to the complexity of the epidemiology [11]. The recent emergence of drug-resistant *C. auris* has exacerbated the burden on healthcare infrastructure. On 25 October 2022, WHO published a list of 19 fungal priority pathogens wherein *Candida auris* and *Candida albicans* are categorized under critical groups, whereas *Candida glabrata*, *Candida tropicalis*, and *Candida parapsilosis* are categorized under high-risk groups [12].

The earliest known *Candida* auris isolate was first identified in South Korea, dating as far back as 1996 [13], and has since rapidly spread across the globe. It has a robust ability to persist on surfaces, and coupled with that, asymptomatic carriers and international travel have contributed to its geographical dissemination and emergence as a major cause of nosocomial outbreaks. However, at that time, it was initially misidentified as a different fungal species, highlighting the challenges in recognizing and characterizing this emerging pathogen. These outbreaks have extended their presence beyond their initial identification in Asia, encompassing countries such as Japan, India, and Pakistan. Additionally, *C. auris* has emerged as a formidable concern in Europe, with reported cases in the United Kingdom and Spain. Across the Americas, the fungus has gained a foothold, affecting countries like Colombia, Venezuela, Panama, and the United States [14]. Perhaps most concerning is its development of multidrug resistance, displaying significant levels of both inherent and acquired resistance to azoles, echinocandins, and amphotericin B, limiting treatment options and increasing healthcare costs. *Candida auris* infections are associated with high mortality rates, particularly among immunocompromised patients, further impacting the global burden of disease. It is essential to highlight that the list of countries grappling with *C. auris* continues to grow, underscoring the pathogen’s ability to traverse borders and establish itself as a global health challenge.

*C. glabrata* is the second most prevalent species in the USA, north-western Europe, and Canada, especially among elderly patients and organ transplant patients. In contrast, *C. parapsilosis* and *C. tropicalis* are more frequently encountered in southern Europe, South America, India, and Pakistan. *C. krusei*, which is the least common among the five main species, tends to occur more often in patients with severe immunodeficiency disorders.

Another less common species, *C. dubliniensis*, is more prevalent among HIV-infected patients. It is important to note that the current epidemiology of invasive candidiasis is significantly influenced by the selection pressure imposed by the use of antifungal medications, both in terms of prophylaxis and treatment. The widespread use of antifungal agents has led to a shift towards non-albicans species, some of which are increasingly resistant to treatment.

A glaring fact to be highlighted here is that around 50% of invasive *Candida* infections go undetected, leading to potential underestimation of the true incidence of these infections. Currently, there is a conspicuous lack of approved diagnostic methods that are both sensitive and precise. Detecting deep-seated organ involvement poses a significant challenge, with blood cultures yielding positive results in fewer than 40% of patients who do not have concurrent candidemia. This underscores a significant gap in our ability to promptly identify and manage these infections within healthcare settings. Undiagnosed *Candida* infections can result in delayed or inadequate treatment, leading to adverse patient outcomes, prolonged hospitalizations, and heightened healthcare expenditures. This places an additional burden on healthcare systems and also endangers patient well-being. In recent years, several non-culture-based techniques have emerged as valuable diagnostic aids, particularly in ruling out invasive candidiasis due to their high negative predictive value. Early initiation of antifungal therapy and effective source control are pivotal factors for survival in patients with invasive candidiasis. Nevertheless, the delay in definitive treatment often occurs due to the limited sensitivity of microbiologic cultures, which are currently considered the gold standard for diagnosis. Hence, there is an urgent need for future research and the development of diagnostic tools that offer both sensitivity and precision. The challenges posed in the diagnosis of *Candida* spp. infections are reviewed elsewhere in detail [15,16].

### 1.2. Challenges in Management of Candida Infections

While superficial mycoses, such as skin and nail infections, are generally manageable, invasive fungal infections present a severe challenge to healthcare systems globally. These infections can range from mild mucosal candidiasis to life-threatening bloodstream infections. The risk factors contributing to the surge in candidiasis cases include HIV infection, immunosuppressive therapy, and prolonged hospital stays. *Candida*-related bloodstream infections are alarmingly common in healthcare-associated settings in the United States and Europe, affecting thousands of patients yearly [7,10].

The COVID-19 pandemic has further exacerbated the global burden of candidiasis. Causal factors such as prolonged admittance in critical care units, prolonged administration of antibiotics and corticosteroids, and micronutrient deficiencies such as that of iron and zinc have led to an increased predisposition to COVID-19-associated candidemia (CAC) [17]. In the context of co-infection, it is of paramount importance to understand the pathogenesis and mechanism of virulence to understand disease progression, especially in the case of co-infections. Reports exist of a direct link between the development of candidiasis and the use of antibiotics and corticosteroids by COVID-19 patients [18]. It is of utmost importance to comprehend the pathogenesis and mechanisms of virulence for a better understanding of disease progression, particularly in cases involving co-infections. To this end, further research is imperative to unravel the intricate mechanisms governing each stage of *Candida* spp. pathogenesis. Although the complete molecular pathophysiology remains incompletely understood, certain factors, such as a weakened immune system, deficiencies in iron and zinc, and the potential for nosocomial and iatrogenic transmissions, increase the susceptibility of COVID-19 patients to candidiasis [17]. Understanding the etiology and pathogenesis of candidiasis is essential for gaining deeper insights into its connection with and transmission in COVID-19 patients.

A study conducted in Cairo, Egypt, demonstrated the effective treatment of a COVID-19 patient with oropharyngeal candidiasis (OPC) through the concurrent administration of miconazole (four times a day) and fluconazole (three times a day), with no distinct side effects [19]. Furthermore, investigating substances capable of impeding biofilm formation may be a novel area of research. Additionally, targeting the host’s micronutrient acquisition for treatment shows potential as a promising avenue of study. Forty-one percent of COVID-19 patients in a hospital-based study were found to be co-infected with *C. albicans*, but the number of azole-resistant pathogens such as *C. auris* also increased. The early diagnosis and treatment of candidiasis were key factors in improving the survival of patients with IC. The survival rate was increased by empirical therapy (66% vs. 44%), the early (within 24 h) prescription of antifungal therapy (56% vs. 38%), and the use of echinocandin (64% vs. 39%) [20]. The magnitude of challenges posed by candidiasis and comparable fungal infections requires a fundamental shift in antifungal treatment research, emphasizing the exploration of secure and preventive approaches like anti-*Candida* vaccines. A primary challenge in the development of vaccines against *Candida* species is the high incidence in immunocompromised population. The immune system’s impairment in these individuals raises concerns both in terms of vaccine efficacy and safety. Unlike viral and bacterial vaccines, which often target surface proteins, fungal pathogens like *Candida* have complex cell walls and lack easily recognizable antigens. Live vaccines generally elicit better immune response, making them more favorable vaccine candidates. However, their use must be cautious due to the potential risk of causing infections in already vulnerable individuals. Conversely, inactivated whole organism and subunit vaccines are considered safer, but they may be less effective in immunocompromised individuals because of their weakened immune systems. Additionally, the diversity of *Candida* species and their ability to cause a wide range of severe infections further complicate vaccine development [7]. Extensive research is being undertaken to enhance adjuvants and vaccine formulations to stimulate more robust protective responses while targeting immune response pathways that may not be compromised [21].

Ensuring the safety and efficacy of such vaccines requires rigorous testing and clinical trials, which can be time-consuming and resource-intensive [22]. Typically, vaccine efficacy assessments begin with inbred mice due to their cost-effectiveness and well-defined immune systems. However, bridging the gap between murine and human immune responses poses a concern, and careful consideration is required when extrapolating efficacy data across species, which poses a great challenge in translating preclinical studies. Furthermore, navigating regulatory processes and scaling up production to meet global demand are substantial logistical hurdles. Converting a vaccine candidate into one approved for human use necessitates substantial financial investments for clinical trials and product manufacturing. Additionally, fungal infections that predominantly affect populations in resource-limited areas often require the involvement of governmental and non-governmental organizations to support vaccine commercialization efforts, ensuring the successful development of an anti-*Candida* vaccine.

Vaccines targeting invasive pathogens constitute a significant advancement in disease prevention, and the development of a successful anti-*Candida* vaccine emerges as a clear strategy for candidemia prevention. *Candida* infections, particularly those caused by drug-resistant strains like *Candida auris*, are on the rise and have a significant impact on healthcare systems globally. With the increasing fatality rate attributed to drug-resistant fungal strains and the growing population of immunocompromised individuals, the development and successful deployment of an effective fungal vaccine hold immense potential. Vaccines offer a promising solution to this problem by potentially preventing *Candida* infections or reducing their severity. By stimulating the immune system to recognize and respond effectively to *Candida* pathogens, vaccines can provide a proactive approach to combatting these infections, potentially mitigating their global impact and reducing the reliance on antifungal medications.

Despite continuing efforts, there is currently no commercially available anti-*Candida* vaccine that has been approved for human use [23,24]. Recently, preclinical and clinical studies of potential vaccine candidates have been published, which report good immunogenicity and a functional response against *Candida* spp. [25,26,27]. With this in mind, the development of an effective and successful vaccine against *Candida* infections could be a crucial step in preventing and controlling these potentially life-threatening fungal diseases. This review outlines the collective efforts made so far in developing diverse categories of *Candida* vaccines, including vaccines undergoing clinical trials. It also addresses the challenges associated with creating a successful anti-candidiasis vaccine in the future.

### 1.3. Rationale for Developing Vaccines against Candidiasis

The treatment of invasive candidiasis in immunocompromised individuals presents a formidable clinical challenge. The current arsenal of antifungal strategies, including polyenes, echinocandins, and azoles, though primary options, suffers from various limitations. These drawbacks encompass restricted efficacy against specific fungal species, adverse effects such as cytotoxicity, hepatotoxicity, allergic reactions, and inflammation, and the emergence of increasing drug-resistant strains. The effectiveness of these antifungal drugs relies on several factors, including the host’s immune system, disease location, severity of infection, and the pharmacokinetics of the drug [28]. The mechanisms of action of antifungal drugs like azoles, polyenes, and echinocandins target specific components in *Candida* and other fungal cells, disrupting their integrity and causing cell death. However, these mechanisms can also lead to adverse effects in humans due to structural similarities or off-target effects. Azoles inhibit the synthesis of ergosterol, a crucial component of the fungal cell membrane. This disruption weakens the fungal cell membrane’s integrity, leading to cell death. However, since human cells also contain cholesterol, which is structurally similar to ergosterol, azoles can have off-target effects on human cells, potentially leading to side effects such as liver toxicity or interactions with other medications. Polyenes, like amphotericin B, form pores by binding to ergosterol, but they can also interact with human cell membranes, leading to kidney damage. Amphotericin B deoxycholate formulations have been associated with severe adverse events, such as nephrotoxicity and infusion-related adverse effects. Echinocandins, by inhibiting beta-glucan synthesis, weaken the fungal cell wall without direct toxicity to human cells, though they can still cause side effects such as gastrointestinal disturbances, elevated liver enzymes, and other adverse effects such as fever, headaches, and allergic responses. Careful monitoring and management of patients are crucial during antifungal therapy to mitigate these adverse effects [29].

Importantly, the emergence of drug-resistant strains of *Candida* species against the majority of antifungal medications and the significant increase in the number of immunocompromised individuals globally (due to various factors like age and increased susceptibility to infections post-COVID-19) emphasize the urgency for innovative antifungal medications, immunotherapies, and antifungal vaccines. In particular, the emergence of *C. auris*, a new drug-resistant species, presents a grave threat to the effectiveness of candidiasis treatment. To address these challenges, experts fervently advocate improved diagnostics, more effective antifungal agents, and immunotherapies against candidiasis [30].

The current situation effectively emphasizes the need for innovative approaches to combat candidiasis. Amidst the call for enhanced therapeutic approaches, the concept of an anti-*Candida* vaccine emerges as a pivotal tool to alleviate the burden of systemic and vaginal candidiasis on a global scale. Such a vaccine holds the potential to significantly reduce mortality rates and, by extension, generate substantial socioeconomic benefits [30]. An anti-*Candida* vaccine, as a preventive measure, holds the promise of not only enhancing public health but also yielding substantial socioeconomic benefits. Firstly, it has the potential to lead to considerable cost reductions within the healthcare sector. *Candida* infections, especially in immunocompromised individuals, often require prolonged hospital stays, costly antifungal medications, and sometimes, surgical interventions. Additionally, vaccines will contribute to resource and time savings by averting the need for the development or discovery of new antifungal compounds.

Additionally, by curbing the incidence of drug-resistant *Candida* strains, an anti-*Candida* vaccine can help mitigate the societal costs associated with antimicrobial resistance. Furthermore, preventing fungal infections in high-risk populations, such as transplant recipients and those with certain medical conditions, can enable individuals to lead healthier lives and contribute more actively to society, ultimately boosting overall productivity and economic growth. The utilization of modern vaccine development technologies, as demonstrated by the rapid and safe development of mRNA-based vaccines against COVID-19, exemplifies an efficient and effective approach to combat fungal infections. These innovations not only enhance our capacity to address fungal diseases but also optimize healthcare efforts and resource utilization. As these efforts converge, a future where candidiasis is under control could become a reality. This would lighten the burden on healthcare systems, which currently bear the weight of managing and treating recurrent cases of candidiasis. Additionally, patients would experience a reduction in the physical and emotional toll caused by the condition. By taking on these challenges head-on and exploring innovative avenues, we pave the way for a future where candidiasis ceases to be a menacing threat.

### 1.4. Description of Current Vaccine Candidates against Candidiasis

**Figure 2:**
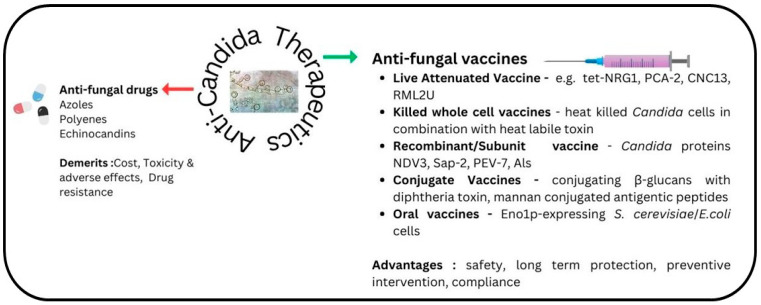
The state-of-the-art anti-*Candida* therapeutics comprise antifungal drugs and potential antifungal vaccines. The advantages conferred by anti-*Candida* vaccines outweigh the antifungal drugs.

The current armamentarium of vaccines targeted against bacterial and viral vaccines is highly successful in reigning the global burden of the respective diseases. Diseases like smallpox and polio have been nearly eradicated, and the incidence of pneumococcal infections, diphtheria, and measles has been significantly reduced. WHO attributes the success against 25 infectious diseases to successful vaccination campaigns. Additionally, the world was a witness to the urgency with which COVID-19 vaccines were developed and administered, leading to a significant reduction in the morbidity and mortality caused by COVID-19 infections l [31,32]. As mentioned earlier, no antifungal vaccine has yet been licensed for human use. The current research is limited to preclinical and clinical testing only. Several vaccine candidates have demonstrated safety and immunogenicity against *Candida* in these studies [25,33]. Hereafter, we describe the state-of-the-art vaccine candidates with potential for tackling *Candida* infections (Figure 2).

#### 1.4.1. Live Attenuated Vaccines

Live attenuated vaccines, a cornerstone of effective immunization strategies against infectious diseases, have demonstrated their potency through successful examples targeting various viruses like polio, measles, mumps, rubella, and influenza and other infections [34,35]. While live attenuated vaccines have historically centered on viruses, recent investigations have revealed their potential in combating fungal infections, particularly candidiasis [34,36].

The principle of live attenuated vaccines revolves around inducing robust immune responses akin to natural disease immunity through the replication of pathogens at the site of infection [10,23,34]. This approach has paved the way for the development of potential vaccines against pathogenic fungi like *Histoplasma capsulatum*, *Blastomyces dermatitidis*, and *Paracoccidioides brasiliensis*. Notably, these vaccines have demonstrated efficacy in stimulating defensive immune responses [10,36].

In the realm of fungal vaccines, a genetically modified *C. albicans* tet-NRG1 strain has been engineered in which the filamentation repressor NRG1 can be overexpressed by doxycycline. The presence or absence of doxycycline (DOX) in the growth medium controls the expression of NRG1, leading to the manipulation of both morphology and virulence. This vaccine construct has shown to protect mice from lethal systemic infection with a virulent fungal strain [37]. Similarly, various attenuated strains of *C. albicans*, such as CNC13 (deleted in the MAP kinase HOG1), RML2U (deleted in the cell wall protein (CWP) gene ECM33 and defective in its interaction with endothelial and epithelial cells), CM1613 (a Mitogen Activated Protein Kinase MKC1 mutant), CNC13 (a MAP kinase HOG1 mutant), and a defective mutant 92′, were able to protect mice from a lethal challenge of *Candida* spp. [30,38]. A genetically engineered *C. albicans* strain PCA-2 is a caspofungin resistant mutant of its isogenic 3153A strain. Immunization of mice with PCA-2 *Candida* triggered an innate immune reaction, with a significant increase in the count of peripheral blood polymorphonuclear cells (PMNs) and significant protection against candidiasis. Further, adoptive transfer of macrophages derived from PCA-2-immunized animals conferred protection against lethal infection in naïve animals [39]. Another attenuated *C. albicans* strain gpi7 effectively protected mice from disseminated invasive candidiasis. In the gpi7 strain, the β-glucan layer of the cell wall is exposed on the surface that facilitates dectin-1 receptor-dependent nuclear translocation of RelB in macrophages, the release of IL-18, and the production of protective antibodies [40].

To combat the problem of safety in immunocompromised hosts, the utilization of viable non-pathogenic fungi *Saccharomyces cerevisiae* has been investigated as a means to serve as carriers for immunogens, encompassing disparate antigens. *Saccharomyces cerevisae* are being explored as vehicles for delivering immunogens in *Candida* infection models. Also, it is being pursued as a vaccine vehicle for pathogens such as the dengue virus, SARS-CoV2, H5N1, *Helicobacter pylori*, *Toxoplasma gondii*, etc. The ease of genetic manipulation in *S. cerevisae* and the potent adjuvant properties of the yeast β-glucan favor this strategy for live attenuated anti-*Candida* vaccine development. Preclinical data have endorsed the activation of DCs and CD4+ T cells, as well as cross-priming CD8+ T cells in response to *S. cerevisae*-based vaccines. In fact, the *S. cerevisiae*-based vaccine vehicles are being pursued as oral vaccines and will be discussed in detail later in the section on oral vaccines [34,41]. “Trained immunity” is an important concept wherein non-specific vaccines like BCG elicit an augmented protective response during subsequent infections, whether caused by the same pathogens or different ones. This heightened defense is primarily facilitated by the innate immune system and provides an enhanced protective response to a secondary infection, caused either by the same or different pathogens, and may lead to beneficial non-specific boosting of immune responses, which may protect against various infections such as *Mycobacterium tuberculosis* and COVID-19 [42,43]. Studies have shown protective innate immune response against pathogens like *C. albicans*, *Staphylococcus aureus*, and *Mycobacterium tuberculosis* upon BCG immunization via epigenetic modifications leading to an enhanced immunological state. BCG-induced enhanced immune response was seen in vivo and in vitro experiments via NOD2-mediated epigenetic changes and histone 3 lysine 4 trimethylation, which ultimately provided increased protection against disseminated candidiasis in SCID mice [44]. Similarly, enhanced protection was observed against an experimental infection caused by an attenuated yellow fever virus vaccine strain via BCG-induced epigenetic reprogramming of innate cells [45]. Therefore, efforts should be made to assess whether BCG vaccination/induction of trained immunity-based vaccines might lower the susceptibility to candidiasis among high-risk individuals. 

However, challenges persist in the translation of these developments into clinical applications. Concerns regarding the stability of attenuated vaccines, vaccine specificity, and the limited use and safety in immunocompromised individuals need careful consideration [30,46]. The key factors withholding the clinical success of the discussed live attenuated vaccines against *Candida* spp., especially in immunocompromised individuals, are as follows: strong immune response that can progress unregulated in immunocompromised individuals; several instances of reversion of avirulent attenuated viral vaccines to virulent forms discouraged the use of live attenuated forms of *Candida* spp.; the constraints of manufacturing, transport, and storage are similar to that of other immunologics. Additionally, in instances of use of a live attenuated vaccine in case of other pathogenic fungi such as *Coccidioides immitis,* no significant benefit was observed in the case of vaccinated and placebo groups [47]. Thus, although the potential of live attenuated vaccines is immense in the case of bacterial infections, in the case of fungal vaccines, prioritizing patient safety is essential due to the inherent risks associated with live fungal infection [48]. 

Conclusively, the road to clinical implementation of antifungal live attenuated vaccines remains intricate, necessitating a balance between efficacy and safety concerns [49].

#### 1.4.2. Recombinant (Subunit) Vaccine

Concerns over traditional live *Candida* vaccines’ complexity and potential unwanted immunological responses have led to the emergence of recombinant protein vaccines. These vaccines leverage genetic engineering to create targeted immunity, often involving the transfer of genes encoding immunogenic antigens. Recombinant protein vaccines are far safer alternatives due to the lack of infectious agents and ease of administration [36]. Agglutinin-like sequence proteins of *Candida* spp. (e.g., Als1p and Als3p) play a crucial role in adhesion and infection [50]. Vaccines based on Als1p and Als3p proteins displayed protection against various forms of candidiasis; indeed, formulations using recombinant Als1p and Als3p demonstrated considerable immunogenic potential and protected against invasive candidiasis [30,36]. The proteins Als1 and Als3, either alone or in conjunction with various adjuvants, have been suggested as potential vaccine candidates for combating invasive candidiasis. When mice were subcutaneously immunized with the recombinant N-terminus of Als1 (rAls1p-N), it protected 50% to 57% of animals against a lethal challenge from *C. albicans* [51]. Positive outcomes have emerged from clinical trials involving NDV-3A, a vaccine based on rAls3p-N. The vaccine candidate NDV-3 consists of recombinant Als3p invasion protein with alum as an adjuvant and was found to be efficacious against candidiasis by inhibiting attachment to epithelial/endothelial surfaces of the Candidiasis. The candidate vaccine NDV-3 displayed significant immunogenicity, eliciting a strong B-cell response and robust T-cell responses in a mouse model, thus successfully preventing both mucosal and hematogenously disseminated candidiasis in mice [25,52]. Further, phase I trials were conducted which included a control group and forty healthy adult subjects that received one dose of NDV-3 containing either 30 or 300 μg of Als3p. Progressing through phase I clinical trials, NDV-3 was found to be secure and effective, generating specific T cells that produce IFN-γ and IL-17A cytokines and also inducing anti-Als3p *total* IgG and IgA1 levels [25] . Another important group of secretory proteins of *C. albicans* that play an important role in fungal cell adhesion, epithelial as well as endothelial invasion, and metabolism are secreted aspartyl proteases (SAP), with Sap2 being the most abundant. When rats were intravaginally or intranasally immunized with recombinant Sap2, whether administered alone or with cholera toxin as an adjuvant, it led to the clearance of *Candida* vaginal infection [53,54]. Amidst the quest for groundbreaking pharmaceuticals and immunomodulatory treatments against *C. dubliniensis* infections, novel approaches such as immunotherapy and in silico investigation for vaccine designs have emerged. SAP proteins within the domain of *C. dubliniensis* have garnered attention as potential vaccine candidates. Computational tools were harnessed to predict epitopes for vaccine development, particularly focusing on SAP proteins [55].

Next, De Bernardis et al. developed a novel vaccine candidate containing a modified version of aspartyl proteinase-2 from *Candida albicans*, enclosed in influenza virosomes (PEV-7). The vaccine elicited a strong antibody in mice and rats following an intramuscular administration. Antibodies were also detected in vaginal fluid after both intravaginal and combined intramuscular and intravaginal administrations in mice and rats. In a rat model of candida vaginitis, PEV7 demonstrated substantial and enduring protection, most likely mediated by antibodies, when administered via the intravaginal route. Furthermore, a repeated-dose toxicological study in rats confirmed the safety of PEV7 [33].

*C. albicans* malate dehydrogenase (Mdh1p) also showed effective results in animals and is being considered for a *C. albicans* vaccine [56]. Additionally, heat shock protein (Hsp90p) and hyphal-regulated cell wall protein1 (Hyr1p) have also shown potential as vaccine candidates [50]. Mycograb, a human genetically recombinant antibody against heat shock protein 90 (rP-HSP90C) was developed. Mycograb, in combination with Amphotericin B, was found to provide complete protection against *C. albicans*, *C. krusei*, and *C. glabrata* but was not approved on grounds of safety and quality [57]. Hyphal-regulated cell wall protein1 (Hyr1) is a GPI-anchored mannan protein present on the fungal cell. Subcutaneous immunization using a recombinant N-terminus Hyr1 (rHyr1-N) with either complete Freund’s adjuvant (CFA) or aluminum hydroxide demonstrated significant protection against *C. albicans*, *C. glabrata*, *C. krusei*, *C. parapsilosis*, and *C. tropicalis* in immunocompetent mice [58].

The use of potent adjuvants in subunit vaccines is a key determinant for vaccine efficacy and conferred protection. Incorporation of the right adjuvant can facilitate multiple advantages such as the targeted delivery of antigens to antigen presenting cells and the formation of antigen depots, aiding cellular chemotaxis, and the stimulation of dendritic cells, B and T cells, etc. They can further activate innate immunity mediators such as Toll-like receptor (TLR) ligands [33,36]. In addition to the traditional adjuvants discussed in the aforementioned section, Wüthrich and co-workers demonstrated the potency of a combination adjuvant, comprising inulin (plant-derived polysaccharide, trade name—Advax) and TLR agonists, in improving the efficacy of a recombinant subunit vaccine *Blastomyces* endoglucanase 2 in a model of respiratory *Blastomyces dermatitidis* infection. The combination adjuvant greatly enhanced the antifungal immunity [59]. In light of these findings, combination adjuvants can also be evaluated for anti-*Candida* vaccine candidates for improving efficacy.

The field of candidiasis vaccine development is progressing with a range of promising recombinant protein candidates. These candidates, targeting distinct antigens, demonstrate immense potential in combating candidiasis and offer avenues for further exploration and development. 

#### 1.4.3. Conjugate Vaccines

Conjugate vaccines, a promising avenue in immunology, involve the strategic fusion of weaker antigens (usually cell wall polysaccharides) with strong immunogenic proteins as a carrier, thereby eliciting robust immune responses. This approach capitalizes on the capacity of polysaccharides to independently stimulate B cells, fostering T-independent immune reactions, while also presenting antigens to T cells through polysaccharide-bound peptides [10]. This synthesis leads to enduring immunity, particularly when protein carriers are affixed to polysaccharides, facilitating binding to MHC molecules and inducing potent T cell responses. (One significant application of conjugate vaccines is their focus on targeting shared polysaccharide epitopes, such as β-glucans found in fungal cell walls.) This innovative strategy holds immense potential for pan-fungal vaccines, crucial for individuals with compromised immune systems and heightened susceptibility to invasive fungal infections. Early attempts included a *C. neoformans* vaccine, merging capsular polysaccharide GXM with tetanus toxoid (TT), yielding significant antibody responses in animals [36]. Further efforts involved conjugating β-glucans with diphtheria toxin (CRM) to counter invasive candidiasis and aspergillosis [50].

The exploration extends to the use of fungal cell wall glycans as alternative protein-based vaccine targets [60]. Mannans and their peptide conjugates emerged as promising *Candida*tes, exploiting the recognition of these glycans by various receptors [30]. The conjugation of mannans with antigenic peptides enhances antigen presentation, yielding effective synthetic glycopeptide vaccines against *C. albicans*. Similarly, β-glucans, crucial components beneath mannans, invoke innate immune responses vital for host defense. Coupling β-glucans with diverse carriers and adjuvants highlights their immunostimulatory potential against candidiasis and aspergillosis [23,61].

Xin and co-workers recently demonstrated a synthetic glycopeptide conjugate vaccine candidate against *C. albicans*. Previous reports by the same group have shown a glycopeptide-tetanus toxoid vaccine candidate that is potentially effective for clinical evaluation [62,63]; however, the glycan component is difficult to synthesize, expensive, and requires significant technical inputs for large-scale manufacturing. This hindered the conventional glycoconjugate vaccines against *Candida*. Additionally, carbohydrate-based vaccines are setback by limited affinity of anti-carbohydrate antibodies as compared to anti-peptide or anti-protein antibodies [64,65]. 

Mimotopes are peptide mimics of glycan epitopes that are easier and cheaper to synthesize and can be explored easily in polyvalent formulations. The glycan part of the glycopeptide vaccine evaluated by Xin and co-workers, β-(Man)3, is ubiquitously expressed across several *Candida* species. Thus, novel peptide mimotopes with structural homology to glycan epitope β-(Man)3 were used in the conjugate vaccine candidate as surrogate epitopes. Specifically, a total of five mimotopes were immunogenic in mice, and out of this, three mimotopes could confer protection in mice against disseminated candidiasis. Furthermore, immunization with three mimotope–peptide conjugate vaccines was also able to induce specific antibody responses, and importantly, protection against disseminated candidiasis in mice. The mechanism of protection via this novel vaccine candidate was indicated to be similar to the protective action of MAbs B6.1-mediated murine neutrophil activity and the B6.1/murine IgG3 isotype variant mediated via recruitment of the host complement system [66]. Xin and coworkers also investigated various mechanistic targets and adjuvants for vaccine formulations; initially, the b-(Man)3-Fba conjugate, combined with dendritic cells (DCs) and complete Freund’s adjuvant (CFA), provided promising mouse protection. However, the use of CFA is not suitable for human use. Alternative adjuvants resulted in weaker immune responses. To boost the immune response and increase vaccine efficacy, the antigen was linked to tetanus toxoid (TT) to form the conjugate (Man)3-Fba-TT. This formulation induced a strong antibody response and a shift from IgM to IgG, indicating development of immunological memory. When adjuvanted with alum or Monophosphoryl Lipid A (MPL), b-(Man)3-Fba-TT showed comparable protection to the original DC/CFA approach [63]. 

In sum, conjugate vaccines stand as a compelling avenue in the fight against fungal infections, leveraging the fusion of antigens with polysaccharides to elicit potent and enduring immune responses. The unique strategy of targeting shared epitopes offers hope for pan-fungal vaccines, benefiting vulnerable populations.

#### 1.4.4. Killed Whole Cell Vaccines

The landscape of vaccine development against candidiasis showcases a range of strategies, with the whole-cell killed vaccine approach standing out for its stability and safety benefits [10]. Whole-cell killed vaccines are stable and non-pathogenic, as compared to live attenuated vaccines, because they cannot revert. The cost of manufacture, ease in handling, and comparative safety make it a preferred choice. This choice is underscored by its simplicity and cost-effectiveness, although challenges in achieving complete protection for mucosal candidiasis have been observed. Diverse avenues have been explored, including the utilization of heat-killed *C. albicans* and a genetically modified toxin as an adjuvant, highlighting potential cross-protection against various fungal infections. Immunization with heat-inactivated *C. albicans* combined with a heat-labile, genetically modified toxin derived from *Escherichia coli*, specifically R192G, as an adjuvant, resulted in a significant level of protection in animal models by intranasal vaccination but did not provide protection via the mucosal route [67]. 

An innovative strategy explored in killed whole-cell vaccine candidate development is the use of *S. cerevisiae* yeast in the form of heat-killed yeast. Subcutaneous immunization with heat-killed *S. cerevisiae* yeast emerges as a promising vehicle for this type of vaccine as it has been shown to confer cross-protection against infections caused by *C. albicans*, *A. fumigatus*, *and Coccidioides posadasii.* Specifically, vaccination with heat-killed yeast cells in a 3, 4, or 6-day schedule conferred protection against a *Candida* challenge via an antibody response directed against glycans common to the cell wall of *Saccharomyces* and *Candida*, in addition to activating cellular responses via Th1 and Th17. Alum was included in the study as an adjuvant, however, it failed to show any significant impact on the strength of immune response [68,69,70]. Yeasts are in fact a standard model for protein expression as they are easy to culture and manipulate and scale up the culture/protein expression. The cellular physiology and molecular biology of *S. cerevisae* is well deciphered. Another advantage of using yeast cells is the ability of yeast surface components to stimulate the human immune response and hence act as an adjuvant. The immunogenicity of *S. cerevisae* is based on the polysaccharides beta-1, 3-D-glucan, and mannan, due to which the dendritic cells are activated and phagocytosed, which, in turn, also generate the danger signals. The Toll-like receptors and mannan receptors are able to recognize the cell wall carbohydrates and aid the adjuvanticity of yeast cells. These factors have aided the use of *S. cerevisae* for developing vaccine candidates via various innovative approaches such as yeast display, whole recombinant yeast cells, heat-killed yeast cells, and preparations of virus-like particles. The yeast-based vaccines are also favorable candidates for oral or edible vaccines [68,69,71]. 

Expanding on the use of heat-killed cells as a vaccine candidate, intranasal application of heat-killed *C. albicans,* plus a heat-labile genetically engineered toxin from *Escherichia coli* as an adjuvant R192G, provided a substantial degree of protection in animal models [67]. Further, a combined vaccine formulation of MV140 and V132 has been tested to prevent both bacterial as well as fungal genitourinary tract infections [72]. MV140 is a polyvalent bacterial preparation based on whole heat-inactivated components used to prevent recurrent urinary tract infections and V132 is heat-inactivated *Candida albicans* vaccine against recurrent vulvovaginal candidiasis. The vaccine combination effectively stimulates human dendritic cells (DCs) to induce IFN-γ and IL-17A-producing T cells, as well as FOXP3+ regulatory Treg cells. Furthermore, MV140/V132 triggers epigenetic reprogramming in human DCs, facilitating the induction of trained immunity. This innovative direction in vaccine research offers a new perspective on fungal infection prevention, as demonstrated by these studies. The complexity of achieving comprehensive protection, especially in mucosal candidiasis, and the interplay between efficacy and toxicity underscore the ongoing challenges in vaccine development. The unique potential of heat-killed yeast-based vaccines to address diverse fungal infections points towards a promising avenue for future research. 

#### 1.4.5. Oral Vaccines

Oral vaccines have significant potential in responding to urgent health challenges due to their convenience, cost-effectiveness, and potential for mass administration. Continued efforts into novel antigenic candidates and adjuvants are crucial in developing effective vaccines to prevent candidiasis, especially in at-risk populations. Significant strides have been achieved through the ingenious utilization of microbial cell surfaces for the display of immunogenic proteins. Notably, the antigen Eno1p from *C. albicans* has been effectively presented on both *E. coli* and *S. cerevisiae* cells, exhibiting promising potential for oral vaccine creation. This transformative approach eliminates the arduous purification process typically associated with traditional vaccine development. Immunization of Enop1 protein in mice via oral or intranasal delivery or through subcutaneous injection resulted in elevated levels of anti-Eno1p antibodies and protected mice against *C. albicans* infection. In one study, Eno1p-expressing *S. cerevisiae* cells protected 60% of the mice against candidiasis when orally administered. A similar effect was observed with *L. casei* cells, where the display of Eno1p yielded 20% protective efficacy in mice [73,74]. While diverse immune adjuvants are available to boost immune response in conventional vaccines, the options for mucosal adjuvants in oral immunization remain limited. This limitation arises because most adjuvants designed for injection cannot withstand the harsh conditions of the gastrointestinal mucosa. Thus, there is a need for novel oral vaccine adjuvants that are both safe and resilient in demanding environmental conditions.

The innovative fusion of immunogenic proteins and microbial cells presents a promising avenue for the development of oral vaccines against candidiasis. The protective effects witnessed in murine models, coupled with the simplicity and speed of the molecular display methodology, showcase its potential in responding to urgent health challenges. The ongoing pursuit of novel antigenic candidates through proteomic analysis holds the prospect of further elevating the effectiveness of these vaccines [75].

#### 1.4.6. Bacterial Ghost Vaccines

Hollow bacterial dead cells with pores that are used to deliver the immunogen or drug to the disease site are called bacterial ghost delivery systems. For the preparation of inactivated vaccines, using formaldehyde or heat treatment compromises the surface structures of the pathogen; however, bacterial ghosts retain the structural features of the antigens (pathogen-associated molecular patterns) displayed on their surface because of the specialized methods based on genetic engineering or chemical treatment. Bacterial ghosts, in fact, have adjuvanticity and can highly boost the host innate immune responses in response to vaccination [76]. Maii and co-workers are pioneers in using the concept of bacterial ghosts in *Candida* vaccine development. They prepared silver and gold nanoparticles using *Candida albicans* ghosts via a modified sponge-like reduced protocol. Sprague-Dawley albino rats aged 4-6 weeks were vaccinated intraperitoneally. The rats were challenged with the hyphal form of *Candida albicans* in artificial ulcers, subcutaneously and intraperitoneally. The authors reported an enhancement in humoral and cellular immunity in the treated rats, along with accelerated ulceration wound healing and controlled inflammation in the ulcer. *C. albicans* ghosts induced the proliferation of all subtypes of white blood cells (WBCs), indicating the activation of cellular immunity, and agglutination tests indicated a positively boosted humoral response. A noticeable outcome was increased systemic IFN-gamma levels, especially even in the *Candida* ghost-administered rats, indicating the benefit of activating phagocytes to mediate the elimination of fungal cells in the absence of any hyperallergenic responses (IgE levels) [77]. Thus, the use of ghost cells is a potentially favorable but unexplored strategy in development of anti-*Candida* vaccines.

### 1.5. Preclinical and Clinical Data on Vaccine Efficacy and Safety

Preclinical and clinical data on vaccine efficacy and safety against *Candida* infections represent crucial stepping stones in the journey toward effective fungal vaccines. As studies continue to unravel the challenges associated with vaccine development against *Candida* and understanding immune response, the ultimate goal is to provide a valuable tool for preventing and mitigating the impact of *Candida*-related diseases, particularly in vulnerable populations. The effectiveness of anti-*Candida* vaccines targeting virulence factors and various forms of *Candida*, such as hyphae and cell wall antigens, has been showcased by mouse models. Diverse formulations are available for these vaccines, including strains that are live and attenuated, recombinant proteins, and glycoconjugates (Table 1). Nevertheless, the challenge posed by the variation among *Candida* species, both genetically and morphologically, is evident and has a major consequence on the success of the vaccine. The majority of previous studies have primarily regarded *C. albicans* as a pathogenic yeast responsible for both primary and secondary infections. However, given its commensal nature, it may potentially maintain a mutualistic relationship with the host, an aspect that has remained largely unexplored. A recent study suggests a mutualistic relationship between *C. albicans* and mice, suggesting that *C. albicans* plays a role in shaping the gut microbiota, influencing metabolism, and bolstering host immunity to the host’s advantage [78]. Designing a vaccine that can cater to a wide array of diseases becomes complicated due to multiple sites of infection and the immune deficiencies prevalent in high-risk groups. Two promising recombinant vaccines, namely, PEV7 and NDV-3, have reached phase I clinical trials in humans. Another important vaccine to reach clinical trials phase 1 is PEV7. It encompasses the recombinant Sap2 protein of *C. albicans* in virosomal formulation. The vaccine was found to be safe and demonstrated specific and effective production of memory B cells.

NDV-3A is the first vaccine to show success in preclinical tests for protecting against diseases caused by both fungal and bacterial pathogens. NDV-3A exhibited heightened antigen-specific titers and cytokine production during a phase 1 trial. After the first dose of immunization, there was an increased expression of anti-Als3p IgG antibodies, in comparison to the control group [25]. Further, an exploratory phase 1b/2a- trial was conducted for NDV-3A against recurrent vulvovaginal candidiasis (RVVC). RVVC is a major concern globally as it affects 138 million women worldwide annually and approx. 492 million are affected once in their lifetime. The vaccine showed a decrease in episodes of vulvovaginal candidiasis for a period of twelve months in women aged under 40 [82]. Nonetheless, in some RVVC cases, therapeutic vaccines might exacerbate the disease due to an overly pronounced inflammatory response. Crucially, the development of a vaccine capable of combatting RVVC holds the potential to pave the way for a vaccine against severe *Candida* infections, even those induced by drug-resistant strains. Thus, it is imperative to continue the progression of the NDV-3A vaccine and provide valuable insights into defining clinically relevant endpoints for the immunotherapeutic management of candidiasis.

## 2. Mechanisms of Immune Protection

### 2.1. Discussion of the Various Immune Responses Elicited by Different Vaccine Types

The immune responses stimulated by fungal vaccines are crucial for advancing vaccine strategies against fungal infections. T cell-mediated responses, supported by various cellular components and soluble components like cytokines, play a central role in combating fungi. Vaccines, especially when used with adjuvants, not only initiate but also enhance immune responses, with some increasing antibody responses, while others primarily intensify T cell responses. Most vaccines boost both types of immune responses. Ensuring the efficacy and safety of antifungal vaccines is essential, especially in individuals with compromised immunity. An ideal antifungal vaccine should be immunogenic and protective without exacerbating immunopathology or worsening underlying diseases.

The understanding of the pathogen infection cycle, structural features on the pathogen, and site of infection are key determinants to balancing the cellular and humoral responses generated by the vaccine candidate under exploration. Inducing a cellular response is preferred for pathogens causing chronic or mucosal infections. Vaccines can utilize attenuated or killed microorganisms, pathogen-specific proteins, or polysaccharide–protein conjugates. Antigen presentation by antigen-presenting cells (APCs) to T cell receptors (TCRs) and the cytokine cocktail generated by pattern recognition receptors (PRRs) is crucial for effective immune response stimulation. Adjuvants like aluminum and calcium salts enhance the humoral response by activating PRRs, while novel adjuvants based on PAMP–PRR interactions aim to enhance specific cellular immunity [83].

Adjuvants play a pivotal role in disrupting immune tolerance during *Candida* infections by enhancing the immune system’s recognition and response to this pathogen. In the context of the human gut, the presence of adjuvants becomes particularly relevant, as *Candida* species are commonly found in the gastrointestinal tract as part of the normal microbiota. By co-administering antigens from *Candida* with adjuvants, the immune system is not only primed to recognize and respond to the pathogen but is also encouraged to break immune tolerance, ensuring a robust and protective immune response. Numerous studies provide substantial evidence supporting the incorporation of pathogen-associated molecular patterns (PAMPs) derived from fungi as potent adjuvants to customize the immune responses elicited by vaccines. For example, various TLRs, Dectin-1, Dectin-2, and Mincle have been recognized for their significant roles, not only in activating innate defense mechanisms but also in regulating the differentiation of T cells toward protective Th1 and Th17 lineages [84]. Further, carbohydrates like glucans, dextrans, lentinans, glucomannans, galactomannans, chitin/chitosan, levans, and xylan derived from fungal components have the potential to be used as antigens as well as adjuvants, as they can stimulate innate cells [85]. This feature presents a promising avenue for investigating molecules with shared structures across different pathogenic fungi as a foundation for designing universally applicable antifungal vaccines. Formulations of fungal vaccines that encompass combinations of fungal PAMPs have exhibited immense potential in mouse models, acting as mediators of precisely tailored antifungal immunity. Among these formulations, glucan particles, enriched with β-glucans and chitin, have demonstrated versatility as carriers for various antigens in antifungal vaccines [86]. A significant hurdle lies in deciphering the components that define effective immunity against diverse pathogenic fungi and in devising strategies to trigger the adaptive immune response effectively, while minimizing any adverse reactions. Clearly, a critical determinant for the success of future vaccine development efforts lies in the judicious choice of adjuvants, which can steer the immune response in a manner that aligns with the specific protective requirements for each fungal pathogen. This approach holds great promise for preventing and controlling Candida infections, especially in individuals with compromised immune systems or those at risk due to medical conditions or treatments that disrupt the delicate balance of gut microorganisms.

Action of vaccine candidates employing cellular immunity mostly rely on Th1 responses; Th1 response is vital for resolving diseases caused by opportunistic pathogens like *A. fumigatus*. Effective vaccination should prioritize directing the immune response towards a Th1 response, inducing key cytokines like IL-12 and IFN-γ. Some protein antigens stimulate the production of IL-12 or IFN-γ after vaccination, suggesting their importance in vaccine efficacy [34].

### 2.2. The Role of Innate and Adaptive Immunity in Protection against Candidiasis

The initial stride in formulating a potent vaccine candidate against fungal infections entails enhancing our understanding of various arms and elements involved in antifungal immune response. Once a deep understanding of the mechanisms governing immune responses to fungal infections is achieved, it will be easy to develop and target new avenues for the success of vaccine strategies. In protection against fungal infections, the roles of innate and adaptive immunity are emphasized, with their relative contributions varying based on the anatomical location of the disease. Innate immunity is crucial on mucosal surfaces, while adaptive immunity becomes essential for protection against mucosal candidiasis in humans. Tissue-specific immunity is evident in systemic candidiasis, where IL-17 plays a role, and Th1 and NK cells are critical. In oral and dermal candidiasis, IL-17A-mediated antifungal immunity is paramount, involving both hematopoietic and non-hematopoietic cells, such as phagocytes, dendritic cells, and mucosal epithelial cells [87]. Tissue macrophages and dendritic cells are pivotal in restricting fungal growth and dissemination, and neutrophils are rapidly recruited to infection sites to combat fungal spores and hyphae [88]. The pivotal phase in triggering an immune response against fungal infections involves the identification of distinct fungal elements known as pathogen-associated molecular patterns (PAMPs) through pattern recognition receptors (PRRs). Various cells of the innate immune system detect fungal components through pattern recognition receptors (PRRs) like TLRs and dectin-1, leading to the activation of immune responses to control fungal infections [89].

Cell-mediated immunity, rather than humoral immunity, is considered the primary protective response against candidiasis [90]. Polymorphonuclear leukocytes and macrophages are essential for protection against candidemia through innate immunity, while CMI through T cells and cytokines predominates in mucosal tissues. The significance of humoral immunity remains inconclusive. CMI is crucial in host defense against mucosal candidiasis in immunocompromised patients and those on corticosteroid therapy. CD4+ cells play a significant role in protection against gastrointestinal *Candida* infections [88]. 

The significance of Th1 cell responses and the production of IFNγ in facilitating the fungicidal activities of both neutrophils and macrophages is well established [91]. The activation and differentiation of IFN-γ-producing Th1 cells is important in vaccine-mediated protection against multiple fungal pathogens. Furthermore, Th17-derived cytokines like IL-17 and IL-22 also play a crucial role in the defense against *Candida* species and in recruiting and activating neutrophils, as well as triggering the activation of epithelial cells and the secretion of antimicrobial peptides [92]. In contrast, cytokines associated with Th2-type immunity have exhibited conflicting roles in the context of a protective host response to *Candida* species [93,94].

Humoral immune mechanisms have been implicated in host defense against *Candida* infections; their contribution to antifungal defense is likely to be more modest compared to cellular mechanisms. Activated complement proteins play a significant role in initiating an appropriate cytokine response but are not capable of directly eliminating *Candida* hyphae in the course of an infection. Studies involving mice deficient in complement factor C3 or C5 display heightened mortality rates due to a compromised ability to resist *Candida* infections or dysregulated inflammatory response [95,96]. A study demonstrated that antibody genes cloned from B cell cultures obtained from patients with *C. albicans* infections exhibited the ability to stimulate opsonophagocytic macrophage activity in laboratory settings. Furthermore, these antibodies were found to confer protection against disseminated candidiasis when tested in live animal models [97]. While the studies are still uncovering the role of the humoral arm of immunity in *Candida* infections, the elicitation of protective antibodies through vaccination may be considered a viable strategy to enhance resistance to such infections [98,99].

The indispensable commensal nature of *Candida albicans* (*C. albicans*) in the human microbiota is not merely a consequence of passive co-existence but rather the outcome of the host’s robust innate and adaptive immune responses, which serve to curtail the proliferation of this potentially hazardous microorganism on epithelial surfaces, a critical defense mechanism against *C. albicans* is the Th17 functional subset of T helper cells. The progressive depletion of these cells in individuals with advancing HIV infection leads to the failure to control fungal infection on oral epithelial tissues, enabling *C. albicans* to unleash its pathogenic capabilities. A significant facet of this pathogenic potential lies in *C. albicans*’ ability to elude host immunity and intensify inflammation and immune activation. Furthermore, HIV infection creates an environment that promotes the overexpression of virulence factors by *C. albicans*, particularly in regard to the secretion of aspartyl proteinases (Saps). These enzymes possess the capacity to degrade vital components of the host’s defense system, such as complement proteins and defensive epithelial proteins [100,101]. The effectiveness of current antifungal therapy over the long term is dependent upon effective collaboration between the host’s immune system and the treatment. Consequently, novel therapeutic strategies aimed at targeting virulence factors and specific immune interventions should be considered. Among these innovative approaches, vaccination holds promise as a potential solution to mitigate the impact of *C. albicans* infections. Targeting antigens that trigger both T cell and antibody responses can be effective in preventing infection. Further, the use of adjuvants that help activate both T cells and B cells, leading to a more robust and protective response, should be considered. Combining multiple antigens from different *Candida* species can create a broader-spectrum vaccine that leads to a robust immune response.

However, immune mechanisms encompassing a range of responses, including those driven by humoral and cell-mediated immunity, or even better, a combination of both these primary components of the acquired immune system, may seem to be a novel and effective approach. The challenge here remains whether the immune response induced by such vaccines can protect against pathogenic *Candida* infections without affecting the role of *C. albicans* as a part of normal microbiota.

## 3. Challenges and Future Directions

### 3.1. Challenges in Developing Effective Vaccines against Candidiasis

The development of effective vaccines against candidiasis is intricate due to challenges rooted in the long-standing co-evolution of *Candida albicans* with humans. The fungus’ presence in the human gastrointestinal tract since birth implies mechanisms of immune evasion via morphological, genetic, and phenotypic adaptability. The development of vaccines against *Candida* species presents a multifaceted challenge, primarily due to the remarkable genetic and morphological variability among these fungi. 

The fungi have the remarkable ability to display morphological and phenotypic plasticity [102]. *C. albicans* is a polymorphic fungus capable of transitioning reversibly between yeast, pseudohyphal, and hyphal forms. This ability is closely tied to its evolutionary adaptation within the human host. The unicellular yeast form *C. albicans* is typically considered a harmless colonizer. However, the transformation to the hyphal form is associated with pathogenesis, as hyphal structures adhere to and invade epithelial cells. Consequently, proteins specific to hyphal or hyphal-associated forms, such as Hyr1, Hwp2, Plb5, and Sod5, have been proposed as potential vaccine targets [103]. Although an advantage of targeting the invasive hyphal antigens/epitopes is highlighted by several research groups, it should be based on the selective targeting of the pathogenic hyphal form, while minimizing the impact on the normal commensal yeast forms and the normal microbiota [104,105]. Nonetheless, thorough evaluation of the utility of hyphal antigens and associated proteins can only lead to a specific vaccine candidate overriding the hindrances due to genetic variations between different *Candida* species. 

Additionally, *C. albicans* undergoes a heritable white-to-opaque phenotypic switch, potentially aiding in immune evasion. Opaque cells are less susceptible to phagocytosis by macrophages and can evade neutrophil killing. Beyond morphological and phenotypic variations, *C. albicans* displays significant genomic plasticity. This includes extensive chromosome rearrangements, aneuploidy, and loss of heterozygosity in response to various stresses, subtelomeric hypervariation, and aneuploidy [106,107]. Such genomic changes enable rapid adaptation to adverse environments by altering the copy number of specific genes on particular chromosomes.

Given the extensive antigenic and genetic diversity within *C. albicans*, a more effective approach to anti-*Candida* vaccine development may involve targeting multiple unrelated virulence-associated antigens simultaneously. Multivalent vaccines, capable o carrying multiple antigens from various strains or serotypes of the same pathogen, could offer a more comprehensive and adaptable strategy against the diverse array of antigens and virulence factors spread over different organs and at different times [108]. This strategic approach involves the amalgamation of well-known immunogenic antigens, and the identification of dominant antigens through computational analysis may play an important role. This approach holds promise in addressing the challenges posed by *Candida* species’ genetic and morphological variations.

Further, the situation is complicated by the development of immune tolerance towards *Candida*, as they are commensal organisms. The diversity in *C. albicans* morphological forms—yeast, pseudohyphal, and hyphal—each contributing to pathogenesis, further complicates vaccine design. Immune tolerance, existing vaccine univalence, and the balance between colonization and infection pose notable challenges in vaccine development. Our complex gut microbiome requires immunological tolerogenic responses to maintain gut homeostasis and prevent chronic inflammation. It is likely that *Candida* species, the most common fungal species in the gastrointestinal tract throughout life, have evolved tolerance mechanisms similar to other gut bacteria like Bacteroides fragilis and certain Clostridia species to regulate the relationship between humans and fungi. One mechanism, likely resulting from the co-evolution of bacterial microbiota, commensal fungi, and the host immune system, relies on the metabolic tryptophan-AhR pathway and 2,3-indoleamine dioxygenase (IDO) [109]. *Candida albicans* induces IDO expression in dendritic cells (DCs) and promotes tolerogenic Treg responses, possibly facilitating its transition from pathogenicity to commensalism.

**Figure 3:**
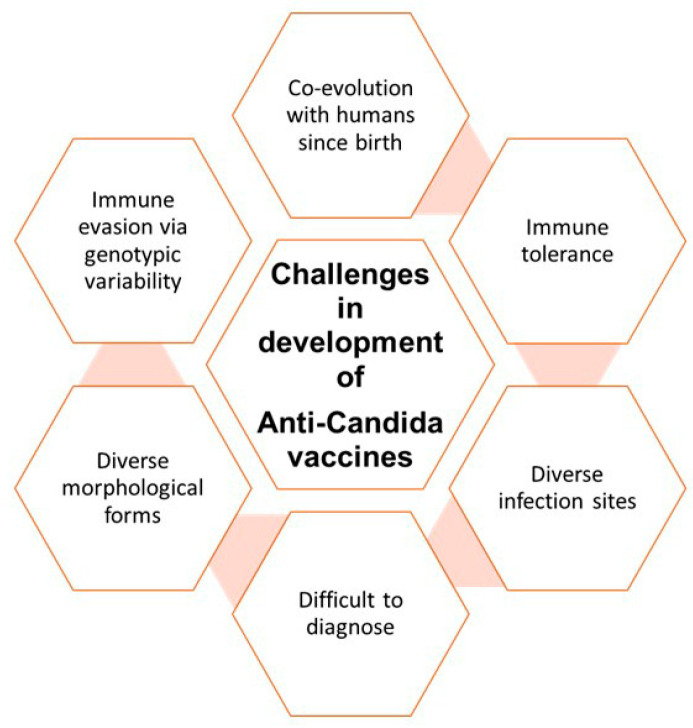
Major challenges in anti-*Candida* vaccine development.

Maintaining immune tolerance toward human gut commensals such as *Candida* represents a challenging task in the way to the development of a potent vaccine against *C. albicans.* The presence of immune tolerance towards *C. albicans*, and potentially other *Candida* species, presents two significant obstacles in the development of vaccines against *Candida*. Firstly, it hinders the development of strong and lasting immunological memory. Secondly, many of the clinical signs of *Candida*-related infections result more from the host immune system’s response than directly by the pathogen [110,111]. Striking the right balance between immunity and tolerance is critical to maintaining gut homeostasis, as breaking host tolerance could lead to unintended consequences, including worsening fungal infections or exacerbating underlying inflammatory or autoimmune conditions. Further, understanding the complex interactions between *Candida* and other commensal microorganisms and their impact on vaccine efficacy may be a significant challenge. The major challenges faced in development of anti-*Candida* vaccines are summarized in Figure 3. Utilizing live attenuated strains in fungal vaccines is challenged by safety concerns, particularly among immunocompromised individuals. Achieving protection in such contexts and addressing potential autoimmunity against commensal fungi is essential. The majority of avirulent strains have faced obstacles preventing their progression to clinical trials. These include factors concerning the possibility of reversion to virulence, infections in people with compromised immune systems, unpredictable immune reactions, risks of horizontal transmission, a lack of information on the safety of vaccines in immunocompromised populations, difficulties with strain stability, genetic modification or mutation stability of these attenuated strains, failure to reproduce results in human volunteers at a clinical trial, and the requirement for stringent safety monitoring and stability during transport. Another significant concern related to conventional vaccines is the uninterrupted preservation of the cold chain, which must be maintained consistently. Clinical trials for live attenuated *Candida* vaccines may exclude immunocompromised individuals, leading to limited data on their safety and efficacy in this population. Ensuring the genetic stability of these attenuated strains over time is also a challenge, and rigorous safety monitoring is essential. As a result, alternative vaccine approaches, such as subunit or inactivated vaccines, are often preferred for immunocompromised individuals in *Candida* vaccine development. Despite these challenges, efforts are underway for antifungal vaccines with alternative routes of administration, lower cost, and adaptability for resource-poor settings, driven by the increasing immunocompromised population and the pressing need to counter fungal infections.

A symbiotic relationship between *C. albicans* and mice influences gut microbiota, metabolism, and immunity. Antifungal drugs induce dysbiosis, emphasizing mutual recognition and protective mechanisms. Immune memory can fail against evolved pathogens, particularly in immunocompromised settings. Efficacy testing with humanized mice models offers a solution. Vaccine strategies must encompass *C. albicans* and non-albicans species. Balancing vaccine effectiveness between healthy and weaker individuals is complex, potentially requiring passive immunotherapy. Adjuvants enhance immune responses, but balancing vaccine safety and effectiveness remains a challenge.

Immunological impairments pose challenges to vaccine effectiveness and safety. Live vaccines exhibit high immunogenicity but carry infection risks. Inactivated whole organism and subunit vaccines enhance safety but may not be effective in immunocompromised individuals. Strategies include enhancing adjuvants and formulations and targeting individuals with robust immune systems. Transitioning from animal studies to human application adds further complexity. Despite several recent concerns about possible risks, alum is still the gold standard for the use of new adjuvants for human use in vaccines. Since the majority of systemic fungal infections gain entry into the host through mucosal surfaces such as the upper respiratory, gastrointestinal, vaginal, or urinary tracts, there is considerable interest in exploring the use of adjuvants for mucosal immunization (via oral, intranasal, and other routes). This approach focuses on stimulating mucosal immune responses, particularly secretory IgA, which is pivotal for delivering antigens effectively to the mucosal-associated lymphoid tissue. This method not only offers a safer and more cost-effective alternative but also simplifies large-scale vaccination efforts. Nevertheless, a significant challenge in creating an efficacious mucosal vaccine lies in the necessity to breach the mucosal epithelial barrier, ensuring efficient antigen presentation to the mucosal immune system, and surmounting the natural tolerance mechanisms at mucosal surfaces. In essence, the judicious selection of adjuvants and delivery systems is paramount to achieving the optimal protective mucosal immune response.

Progress in vaccine development has been notable, yet several challenges persist. Key issues include potential differences in vaccine efficacy between animal models and humans, limited vaccine persistence and efficacy, possible vaccine-related toxicity, lack of standardized manufacturing processes, resource-intensive clinical trials, and the complexity of ensuring vaccine stability during production, transportation, and storage. Enhanced comprehension of *C. albicans*–host interaction mechanisms is pivotal for identifying new targets. Preclinical trials should encompass various animal species, diverse host statuses, and experimental parameters like susceptibility and dosing. Real-time feedback in clinical trials is essential for determining dosing regimens based on safety, pharmacokinetics, and therapeutic effects. The critical need to assess vaccine–drug synergy and overcome immune response obstacles in immunocompromised individuals underscores the ongoing quest for a systemic *C. albicans* infection.

### 3.2. Potential Directions for Future Research

The advancement of an effective anti-*Candida* vaccine demands innovative strategies that encompass diverse facets of research [112]. The ideal vaccine necessitates heightened immunogenicity, broad-spectrum protection, addressing superficial and bloodstream infections, and effectiveness in immunocompromised individuals. As conventional approaches targeting a single antigen prove limited, the current focus leans towards multivalent formulations composed of multiple antigens from various strains. This strategy not only mirrors successes in other vaccine domains but also addresses the challenges posed by *C. albicans*’ virulence factors. In lieu of univalent vaccines, the notion of targeting multiple virulence-associated antigens concurrently has emerged to prevent the emergence of “escape mutants” and a greater specificity against the pathogen. This would help preserve the commensal, and potentially beneficial, aspects of this gut inhabitant. One strategy may be to design a multivalent vaccine using antigens showing effective results in univalent vaccines such as Als3 and Sap2. Such multivalent vaccines would address the challenge of genetic and phenotypic diversity shown by *Candida*. The antigenic targets can encompass various *Candida* strains and different forms of the fungus, such as yeast and hyphal forms. Combining major antigens associated with critical *C. albicans* virulence or biological functions can have a synergistic effect on immune responses. This approach can broaden the range of protective antibodies generated by the immune system and decrease the likelihood of fungal immune evasion. By targeting multiple aspects of the fungus’ virulence and biology simultaneously, a more comprehensive and robust defense can be mounted against *C. albicans* infections [80].

Trained immunity, induced by certain vaccines, is mediated by innate cells like monocytes, macrophages, or NK cells. Traditionally, the focus has been on adaptive immune responses, but recent research has unveiled the potential of the innate immune system’s memory. Recognizing the potential of harnessing trained immunity, induced by specific vaccines, could be of paramount importance, particularly given *Candida*’s genetic diversity. A significant portion of research in this domain has focused on inducing trained immunity using bacterial products like lipopolysaccharide (LPS) or Bacillus Calmette–Guérin vaccine (BCG), which can activate distinct trained immunity pathways guarding against subsequent infections. This concept holds potential in reducing candidiasis vulnerability. Some of the earliest instances of trained immunity were demonstrated in murine studies through low-dose *Candida* spp. infections in T and B cell-depleted animals, which exhibited protection against subsequent lethal fungal infections. This immune response was mediated by the fungal cell wall component β-glucan and relied on functional circulating monocytes [113,114]. The significance of trained immunity could potentially open up an important avenue for achieving cross-protection against various other infections. Systemic infection of mice with avirulent *C. albicans* provided protection against virulent strains [39]. The protective effect was mediated by macrophage-like cells and was found to be non-specific, as it equally extended to cross-protection against *C. tropicalis* and *Staphylococcus aureus* [42]. Promising antigens like β-glucan have been explored in this context, as similar effects were observed, mediated by increased secretion of cytokines like TNF-α and IL-6 from the β-glucan trained monocytes [30]. A preliminary study was conducted to explore the feasibility of employing β-glucan as a vaccine in humans. This involved the oral administration of β-glucan, followed by the assessment of innate immune reactions in PBMCs of healthy volunteers that were later stimulated with *C. albicans in vitro* [115]. Unfortunately, the findings yielded less favorable outcomes in human subjects. Further studies using different administration routes are still worth exploring to assess the role of β-glucan as a vaccine candidate to induce trained immunity against subsequent *Candida* infections. By inducing trained immunity through exposure to avirulent *Candida* species or antigens, the body can develop a heightened state of readiness to combat subsequent *Candida* infections. This approach offers several advantages, including the capacity to provide broad-spectrum protection against different *Candida* strains and the ability to enhance the immune response in immunocompromised individuals [116].

Antigens derived from *Candida* molecules show promise for animal model and clinical trial experimentation. Preventive vaccines for superficial candidiasis are of particular interest, targeting recurrent vaginal candidiasis and denture stomatitis. Recombinant protein-based vaccines, rAls3p-N (NDV-3) and rSap2t (PEV7), hold potential against recurrent vaginal candidiasis and even antibiotic-resistant *S. aureus* infections. Focus also extends to systemic vaccines against invasive candidiasis, potentially synergizing with antifungal drugs. MNs or BGs, combined with appropriate adjuvants, is a promising area for the development of pan-fungal vaccines [117]. Phylogenetically conserved antigens like MNs and BGs are found in the cell walls of many fungal species. These components exhibit common structural elements and functions across various fungal pathogens. For example, MNs are critical for cell wall integrity in numerous fungi, making them attractive targets for pan-fungal vaccines. Further, these antigens can induce immune responses with cross-reactivity against multiple fungal species. Pan-fungal vaccines are cost-effective and efficient, offering a broader spectrum of protection against fungal infections like candidiasis, aspergillosis, and pneumocystosis. A “pan-fungal” peptide vaccine, NXT-2, was developed by utilizing a previously identified recombinant pan-fungal protein (NXT-2) in multiple models of invasive fungal disease. Vaccination with NXT-2 significantly enhanced immunogenicity and showed protection in severely immunosuppressed animal models of aspergillosis, candidiasis, and pneumocystosis, compared to controls, thus suggesting that immunization with a pan-fungal vaccine could provide broad, cross-protective antifungal immunity for at-risk individuals, potentially addressing a critical gap in fungal infection prevention [81]. However, challenges such as selecting the right conserved antigens, designing effective formulations, and achieving desired immunogenicity are the key for a successful vaccine candidate.

In crafting the ideal vaccine, the emphasis lies on high immunogenicity, encompassing protection against various fungal pathogens and conditions. The pursuit of a pan-fungal vaccine capable of addressing both superficial and bloodstream infections for individuals with compromised immunity is of paramount importance. The utilization of DNA polymerase subunit knockouts to generate whole-cell vaccines shows promise. 

A pan-fungal vaccine strategy is essential, supported by DNA polymerase subunit knockouts in *C. albicans*, promising systemic candidiasis protection, even in non-albicans species. These advances mirror those directed against viruses and bacteria, fostering hope for multivalent vaccine development, and broadening protecting immunity against multiple infections.

Resolving the intricacies of host–fungus interaction, particularly concerning cell wall glycans, offers potential for novel treatments [118]. Amidst the challenges of drug-resistant fungal pathogens, efforts in diagnostic and therapeutic innovation continue. Ongoing clinical trials targeting high-risk groups, including the immunocompromised, strive to combat emerging fungal threats [119]. Understanding antifungal drug resistance mechanisms through chemical genomic approaches aids treatment strategies. The exploration of diverse chemical libraries could lead to targeted antifungal solutions [120]. It is vital to keep in mind that the vaccine being developed must not disrupt *Candida* species in their advantageous commensal state because doing so could impede the host’s ability to grow. These therapies ought to be similarly effective in those with compromised immune systems. Instead of depending on inbred animals, vaccine candidates can be tested during the preclinical stage using humanized and immunodeficient mouse models like SCID or nude mice, and further validated through bigger animal models. A whole-cell vaccine that is specially tailored to target the pathogenic form offers distinct advantages in instances where antigenic peptide-based vaccines could find it difficult to differentiate between commensal and pathogenic *Candida* forms. Given the ease of attenuated and whole-cell vaccines, highly stable and antigenic live attenuated strains of *C. albicans* should be investigated, as they could potentially offer broad-spectrum protection.

Further, oral vaccines offer numerous advantages, such as ease of administration, cost-effectiveness, and potential for mass immunization. Such vaccines are increasingly attracting interest because of their convenient mode of delivery, reduced invasiveness, typically enhanced safety profile, and cost-effectiveness compared to injectable vaccines. The development and utilization of oral vaccines are key in addressing urgent health challenges and are exemplified by recent advances in the COVID-19 pandemic. The identification of unique antigenic proteins within Candida species and the role of cell wall glycans and other virulence factors in Candida may lead to the development of a novel oral vaccine candidate.

Further, addressing the challenges in developing effective candidiasis vaccines requires thorough exploration for adjuvant optimization. The strategic choice of potent adjuvants and delivery systems is instrumental in steering immune responses towards desired outcomes, an indispensable facet of successful vaccine development. It is important here that we explore novel, biocompatible, and potent adjuvants such as phytocompounds (ginsenosides like Rg1, Re, and Rd) or strategies such as combination adjuvants against *C. albicans* for a more effective and cost-efficient *Candida* vaccine [59,121]. 

An emerging new area which can accelerate the development of an anti-*Candida* vaccine is that of in silico epitope prediction using immunoinformatic approaches—the development and use of algorithms and tools for predicting B and T cell epitopes. The pathogen genome can be analyzed to identify potential antigenic proteins and the epitope sequences. Protein 3D structures and antigenic features can also be screened using a structural and sequence homology search, followed by protein–protein interaction analysis and docking. These approaches can increase the efficiency of a vaccine candidate search, especially in terms of cost and time taken for identifying potential epitopes [122]. Akhtar and coworkers recently demonstrated the use of immunoinformatic approaches in predicting a potent vaccine candidate against *Candida tropicalis.* They identified eleven conserved antigenic but non-allergic and non-toxic epitopes on secreted aspartic protease 2 protein. Additionally, they paired the epitopes RS09 (LPS peptide mimic/TLR agonist), flagellin sequences, and PADRE sequence to create a vaccine candidate that can potentially elicit humoral and cell-mediated immune responses. The construct was also indicated to be non-homologous to any human protein and hence potentially safe [79]. 

Collectively, these multidimensional pathways chart the course for effective anti-*Candida* vaccine development. Innovative strategies such as multivalent formulations, trained immunity, and exploring for immunogenic but safe *Candida* antigens and/or adjuvants underscore the possibilities. Pioneering experimental initiatives that focus on particular infections and specific populations, along with a detailed understanding of drug resistance mechanisms, drives the journey forward. While there is certainly a requirement for safe and efficient vaccine, the obstacles are significant in terms of both conceptual understanding and technical aspects in developing successful vaccines against conditions like candidiasis and other fungal infections. Nonetheless, the comprehensive approach, along with the pursuit of an effective anti-*Candida* vaccine, holds the potential to combat these fungal infections and enhance global health outcomes.

## 4. Conclusions

Candidiasis—the diverse spectrum of fungal infections caused by various *Candida* species—is a substantial global health challenge. Increasing reports of multidrug resistance in *Candida* species and the expanding immunocompromised population underscore the urgency for inventive approaches like a novel prophylactic vaccine. However, an anti-*Candida* vaccine is yet unrealized due to the challenges posed by the variability and adaptability of *Candida* species, pre-existing immunological tolerance, and impaired adaptive immunity in certain patients. The advantages of an anti-*Candida* vaccine are likely to benefit a large socio-economic demographic and represent long-term measures to reduce the disease incidence. The authors endorse a multifaceted strategy to facilitate vaccine development against *Candida* spp.—screening various pathogenic species’ and strains’ of *Candida* genome and proteome for novel, safe and potent epitopes, identifying stable and antigenic strains, the optimization of vaccine formulations with a special focus on novel adjuvants, and rigorous clinical trials to ensure a safe and efficacious vaccine. Stress is also to be laid on meticulous animal model selection, stability assessments, and safety protocols as a crucial step before entering clinical phases. Additionally, striking a balance between cellular and humoral responses based on pathogen and infection site is crucial for safe and effective antifungal vaccines, especially for immunocompromised individuals. Furthermore, harnessing the emerging insights into trained innate immunity, multivalent vaccines and pan-fungal vaccines offers promising prospects. The enlisted strategies can not only facilitate tackling candidiasis but also potentially provide cross-protection against other opportunistic infections. Collaborations are warranted between researchers, healthcare providers, regulatory bodies, and industries to pave the way to the successful development of a novel anti-*Candida* vaccine.

## Figures and Tables

**Table 1:** List of various vaccine candidates in development against candidiasis.

Vaccine Category	Description	Clinical Trial Phase	Preclinical and Clinical Data on Vaccine Efficacy and Safety	Reference
Live Attenuated	Genetically modified *C. albicans* tet-NRG1 strain engineered for hyphal growth control; PCA-2, CNC13, RML2U, and tet-NRG1 strains effective against infections; *Saccharomyces cerevisiae* as a safe carrier for vaccines.	Preclinical	Genetically modified *C. albicans* effectively protect against candidiasis in mouse models. *Saccharomyces cerevisiae* as a vaccine carrier demonstrates safety and immune response.	[30,50]
Recombinant (Subunit)	Utilization of recombinant proteins (Als proteins, Sap-2, and NDV-3); PEV7 containing modified aspartyl proteinase-2; strong antibody responses and protection in animal models.	Phase I/II	Phase I/II trials show stimulation of strong antibody and specific T cell responses. PEV7 and NDV-3 demonstrate enhanced efficacy in animal models.	[10,33,36]
Conjugate	Fusion of potent antigens with polysaccharides; targeting shared polysaccharide epitopes (β-glucans; mannans); protection against candidiasis and aspergillosis.	Preclinical/Phase I/II	Preclinical studies demonstrate protection against fungal infections. Phase I/II trials show immunostimulatory effects and potential for pan-fungal vaccine.	[30,50,63]
Killed Whole-Cell	Whole-cell killed vaccine approach (heat-killed *C. albicans*); protection against systemic candidiasis and other fungal infections.	Preclinical/Phase I/II	Preclinical studies show protection against systemic candidiasis. Phase I/II trials indicate potential for broad-spectrum fungal protection.	[10,68]
Oral	Display of immunogenic proteins on microbial cell surfaces; Eno1p antigen from C. albicans displayed on *E. coli* and *S. cerevisiae* cells; protection against candidiasis.	Preclinical/Phase I/II	Preclinical studies demonstrate protection against candidiasis. Phase I/II trials show efficacy protection.	[73,74]
Multi-Epitope	In silico design of a multi-epitope vaccine against *C. dubliniensis*; potential immunogenicity; further in vivo investigations needed.	Preclinical	Immunoinformatic approach identifies eight epitopes for a C. dubliniensis vaccine candidate with potential immunogenicity. Further in vivo studies required for safety and efficacy assessment.	[79]
β-(1,3) Glucan	Synthesis of linear β-(1,3) glucan-CRM197 conjugate; elicits uniform IgG response in mice; exploration of protective potential.	Phase I/II	Linear β-(1,3) glucan-CRM197 conjugate induces a uniform IgG response in mice, demonstrating potential for *C. albicans* epitope coverage. Efforts are underway to explore protective potential.	[60]
Proteome-Wide Subunit	Identification of immunodominant epitopes in hyphal proteins; broad applicability; RS09 adjuvant inclusion.	Preclinical/Phase I/II	Proteome-wide immunoinformatic strategy to select 18 epitopes for *C. albicans* subunit vaccine. Epitopes are conserved and bound to multiple HLA class II alleles. RS09 used as an adjuvant enhances immune response.	[80]
Pan-Fungal Recombinant	Development of NXT-2, a pan-fungal recombinant protein vaccine; effectiveness against aspergillosis, candidiasis, and pneumocystosis; cross-reactivity.	Preclinical/Phase I/II	NXT-2 demonstrates effectiveness against multiple fungal infections in animal models. It elicits strong immune responses and shows cross-reactivity with various fungal pathogens. Further research needed for evaluation in humans.	[81]

## Data Availability

No new data were created or analyzed in this study. Data sharing is not applicable to this article.

## Abbreviations

APCs: Antigen-Presenting Cells; BCG: Bacillus Calmette-Guérin; CMI: Cell-Mediated Immunity; TLRs: Toll like receptors; RML2U: Regulated Morphogenesis Locus 2 Unstable; WHO: World Health organization.
